# XGBoost-based machine learning model combining clinical and ultrasound data for personalized prediction of thyroid nodule malignancy

**DOI:** 10.3389/fendo.2025.1639639

**Published:** 2025-07-29

**Authors:** Wenhan Li, Yajing Zhou, Ziyu Luo, Miao Tan, Rui Yin, Jianhui Li

**Affiliations:** ^1^ Department of Surgical Oncology, Shaanxi Provincial People’s Hospital, Xi’an, Shaanxi, China; ^2^ The Third Affiliated Hospital, School of Medicine, Xi’an Jiaotong University, Xi’an, Shaanxi, China; ^3^ Department of Thyroid and Breast Surgery, The First Affiliated Hospital of Henan Polytechnic University (The Second People’s Hospital of Jiaozuo City), Jiaozuo, Henan, China; ^4^ Department of General Surgery Ward 1, Hospital of Ningshan County, Ankang, Shaanxi, China

**Keywords:** thyroid nodules, machine learning, XGBoost, diagnosis, web-based calculator

## Abstract

**Purpose:**

Thyroid ultrasound is a primary tool for screening thyroid nodules (TNs), but existing risk stratification systems have limitations. Nowadays, machine learning (ML) offers advanced capabilities to handle high-dimensional data and complex patterns. This study aimed to develop an ML model integrating clinical data and ultrasound features to improve personalized prediction of TN malignancy.

**Methods:**

Data from 2,014 patients with TNs (2018.01–2024.01) were retrospectively analyzed, with 1,612 in the training set and 402 in the test set. Features included demographic, ultrasound, and thyroid function indices. Random Forest (RF) and Lasso regression were used for feature selection. Furthermore, six ML models (KNN, Logistic Regression, RF, Classification Tree, SVM, and XGBoost) were developed and validated via 10-fold cross-validation, evaluating performance using area under the receiver operating characteristic curve (AUC), accuracy, sensitivity, specificity, calibration curves, and decision curve analysis (DCA).

**Results:**

17 variables were influential factors for diagnosing TNs. All six models exhibited satisfactory predictive performance, with their accuracy ranging from 0.761 to 0.851 and AUC from 0.755 to 0.928. Among them, the XGBoost model demonstrated the best performance, achieving an AUC of 0.928, accuracy of 0.851, sensitivity of 0.933, and specificity of 0.650. Calibration curves showed strong agreement between predicted and observed malignancy probabilities, and DCA indicated net clinical benefit across a wide risk threshold range (0.2–0.9). Additionally, we have developed the model as a web-based calculator to facilitate its practical application.

**Conclusions:**

The XGBoost model effectively integrates multi-modal data to predict TN malignancy, offering improved accuracy and clinical utility.

## 1 Introduction

In recent years, the increasing adoption of high-resolution imaging systems has led to a steady annual rise in the diagnosis of thyroid tumors. Thyroid ultrasound is regarded as the optimal screening method for thyroid nodules (TNs) due to its cost-effectiveness, non-radiative property, and high-resolution capability (1, 2). Many ultrasound-based risk stratification systems have been widely applied in clinical practice, which are designed to quantify the probability of malignancy and thereby guide clinicians in subsequent treatment decisions (3–5). Nonetheless, these systems have notable limitations: they provide broad estimates of malignancy risk (e.g., ACR-TIRADS 4 defines a malignancy risk spanning 2% to 90%) and exhibit inter-system variability when classifying identical TNs—which should not be ignored (6, 7). Previously, we have constructed a nomogram diagnostic model based on the ultrasonic features and patients’ clinical data, which has achieved good predictive performance (area under the receiver operating characteristic curve (AUC): 0.882, Accuracy: 0.815) (8). However, like traditional statistical models, this nomogram relies on strict assumptions (e.g., linearity, low dimensionality), limiting its ability to capture complex feature interactions and high-dimensional data patterns.

As artificial intelligence (AI) advances, machine learning (ML) has emerged as a pivotal technique for analyzing large-scale, high-dimensional datasets, addressing limitations of traditional statistical methods in capturing complex patterns (9–11). The primary superiority of ML compared to traditional statistical approaches is its capacity to independently discover and learn intricate patterns embedded within datasets, a process that operates without reliance on pre-established model assumptions. Unlike conventional approaches, which rely on pre-established assumptions, ML algorithms independently identify intricate patterns within data through iterative learning and optimization. This process enhances a model’s generalization ability, enabling it to adapt effectively to unseen data and real-world clinical complexities (12). Although radiomics-based TN diagnostic models have been reported, their biological interpretability remains poor (13, 14). Moreover, patient-specific factors such as age, gender, and thyroid function significantly influence diagnostic outcomes. Against this backdrop, we developed an ML model that integrates preoperative clinical data and ultrasonographic features to predict individual TN malignancy probabilities, aiming to facilitate more accurate, non-invasive, and personalized clinical management.

## 2 Materials and methods

### 2.1 Data collection

A total of 2,783 consecutive patients with suspicious TNs were enrolled between January 2018 and January 2024. In accordance with the ethical guidelines of the Declaration of Helsinki and the local regulations on medical research involving human subjects, our institutional ethics committee explicitly granted a waiver of informed consent for this study. (Approval No.: 2024-013). All subjects underwent fine-needle aspiration (FNA) and thyroid function tests before any treatment initiation. Ultrasonography was performed by an experienced radiologist (with ≥10 years of thyroid imaging expertise). Patients were excluded if they had a history of thyroid surgery, indeterminate pathological diagnosis, or incomplete clinical data.

### 2.2 Data pre-processing and feature selection

To enhance predictive performance and mitigate overfitting, we employed random forest (RF) for feature selection to identify key predictors of malignant nodules. The process was as follows: (I) Suppose the RF consists of K trees. Each tree requires a specific number of sample sets for training. These sample sets are randomly generated through the Bagging method. (II) M is used to represent the number of features. For each tree node, m features are randomly chosen, where m should be significantly smaller than M. The optimal splitting approach is computed based on these m features using the Gini coefficient. (III) Through Bagging, we used out-of-bag (OOB) error evaluate the model’s generalization ability. 500 decision trees were constructed, and 3 variables were randomly selected at each decision tree node. Following the above - mentioned method, RF was used to screen variables based on feature importance. In addition, given the outstanding feature selection capabilities of the least absolute shrinkage and selection operator (Lasso), we also performed Lasso regression and compared its results with RF. Lasso uses an L1 norm penalty to shrink irrelevant feature coefficients to zero, with the optimal λ (0.0261) determined via cross-validation at 1 standard error from the minimum error.

### 2.3 Development of ML-based models

Six ML algorithms—K-nearest neighbor (KNN), logistic regression (LR), random forest (RF), classification tree (CT), support vector machine (SVM), and extreme gradient boosting (XGBoost)—were developed to predict TNs malignancy. These models underwent internal validation via 10-fold cross-validation. Subsequently, their discriminative ability was evaluated using metrics including AUC, accuracy, sensitivity, specificity, F1 score, positive predictive value (PPV), negative predictive value (NPV), DeLong test, and. Furthermore, calibration curves and decision curve analysis (DCA) were applied to assess the models’ clinical utility. Finally, we developed a web-based calculator using the optimal model. This calculator is developed using the R Shiny framework. Its core computational logic is encapsulated into standalone functions, which are rigorously tested through unit tests implemented with the ‘testthat’ package. Additionally, automated interactive testing is performed using ‘shinytest2’ to validate the correct responses to user inputs and outputs. Finally, the application is deployed in a controlled environment to ensure reliable and consistent results.

### 2.4 Statistical analysis

Statistical analyses were performed using SPSS (Version 26.0) and R software (Version 4.3.2). T-tests and chi-square tests were used to analyze demographic and clinical characteristics of all included patients. R packages—”Boruta”, “caret”, “calibrate”, “class”, “e1071”, “ggplot2”, “glmnet”, “kknn”, “kernlab”, “partykit”, “pROC”, “randomforest”, “reshape2”, “rms”, “rpart”, and “xgboost”—were utilized for model development and validation. Performance metrics including AUC, accuracy, sensitivity, specificity, F1 score, positive predictive value (PPV), and negative predictive value (NPV) were calculated to evaluate and compare models. Finally, discriminative ability was quantified via calibration curves and decision curve analysis (DCA). A two-sided *p* < 0.05 was considered statistically significant.

## 3 Results

### 3.1 Clinical and ultrasound characteristics

**Figure 1:**
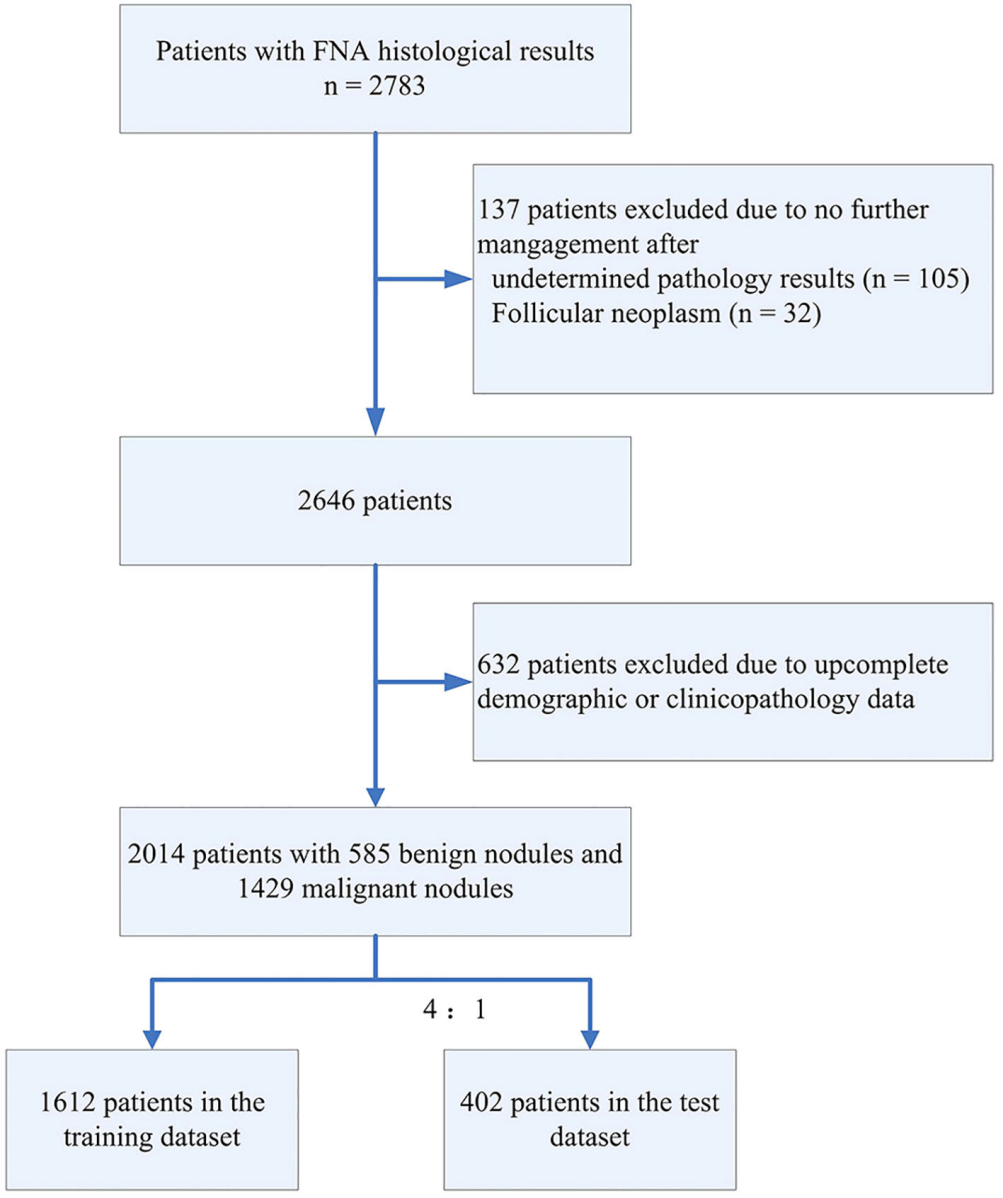
Data processing flowchart.

In total, 2783 patients with FNA results were included in our study. After data screening, a total of 2,014 cases (mean age 46.89 ± 12.55, 527 male) with complete information were included. Those research subjects were randomly divided into the training set (1612 cases with 468 benign nodules and 1144 malignant nodules) and the test set (402 cases with 117 benign nodule and 285 malignant nodules). The flowchart depicting the study is presented in **Figure 1**. **Table 1** summarized characteristics of those patients. Overall, demographics analysis showed that the proportion of male patients and the BMI index with malignant TNs are significantly higher than those of patients with benign TNs, while the age of onset is significantly lower than that of patients with benign TNs (All *p* < 0.05). Laboratory test demonstrated that compared with patients with benign TNs, patients with malignant nodules have lower levels of TPO Ab, while their levels of fT4 and fT3 are relatively higher (All *p* < 0.05). Regarding the ultrasonic characteristics, the results of our analysis showed that there are significant differences in nodule size, margin, extrathyroidal extension, halo, composition, echogenicity, calcification pattern, suspicious LNM, and aspect ratio between benign and malignant thyroid tumors (All *p* < 0.05).

**Table 1:** Baseline characteristics of included patients.

Characteristics	Overall n = 2014	Benign n = 585	Malignant n = 1429	*P* value
Demographics				
Gender				
Male	527	122	405	<0.01^a^
Female	1487	463	1024	
Age (mean ± SD)(year)	46.89 ± 12.55	51.79 ± 12.68	44.89 ± 11.93	<0.01^b^
BMI	24.22 ± 3.69	23.94 ± 4.04	24.33 ± 3.54	0.04^b^
Laboratory test				
TSH (mean ± SD) (uIU/mL)	3.59 ± 6.10	4.06 ± 7.87	3.40 ± 5.15	0.06
TPO Ab (mean ± SD) (IU/mL)	63.86 ± 161.96	96.69 ± 206.99	50.42 ± 137.23	<0.01^b^
TG Ab (mean ± SD) (IU/mL)	86.23 ± 216.13	220.36 ± 9.11	85.65 ± 214.44	0.85
fT4 (mean ± SD) (pmol/L)	15.87 ± 3.83	15.50 ± 3.78	16.02 ± 3.85	<0.01^b^
fT3 (mean ± SD) (pmol/L)	5.01 ± 1.22	4.82 ± 1.03	5.08 ± 1.28	<0.01^b^
Tg (mean ± SD) (ng/mL)	90.25 ± 453.22	95.19 ± 327.47	88.23 ± 495.67	0.75
Imaging				
Nodule size (mean ± SD) (cm)	1.25 ± 1.12	1.56 ± 1.44	1.12 ± 0.94	<0.01^b^
Margin				
Regular	356	237	119	<0.01^a^
Irregular	1658	348	1310	
Extrathyroidal extension				
Yes	357	9	348	<0.01^a^
No	1657	576	1081	
Halo				
Absent	1815	502	1313	<0.01^a^
Complete	81	71	10	
Incomplete	118	12	106	
Composition				
Cystic or spongiform	6	3	3	<0.01^a^
Cystic and solid (Cystic≥50%)	19	15	4	
Cystic and solid (Solid≥50%)	102	62	40	
Solid	1887	505	1382	
Comet tail artifacts				
Yes	10	3	7	0.947
No	2004	582	1422	
Echogenicity				
Hyperechoic or isoechoic	19	13	6	<0.01^a^
Mix	120	76	44	
Hypoechoic	1843	490	1353	
Very hypoechoic	32	6	26	
Calcification pattern				
No calcification	632	235	397	<0.01^a^
Macrocalcification	198	99	99	
Macro and microcalcification	114	33	81	
Microcalcification	1070	218	852	
Vascular distribution pattern				
Avascularity	810	234	576	0.07
Peripheral vascularity	477	124	353	
Mainly central vascularity	476	132	344	
Mixed vascularity	241	85	156	
Suspicious LNM				
Yes	341	28	313	<0.01^a^
No	1673	557	1116	
Aspect ratio >1				
Yes	1096	197	899	<0.01^a^
No	918	388	530	

a Using χ2 test for this statistic.

b Using two-sample t-test for this statistic.

### 3.2 Screening of risk factors for thyroid cancer

**Figure 2:**
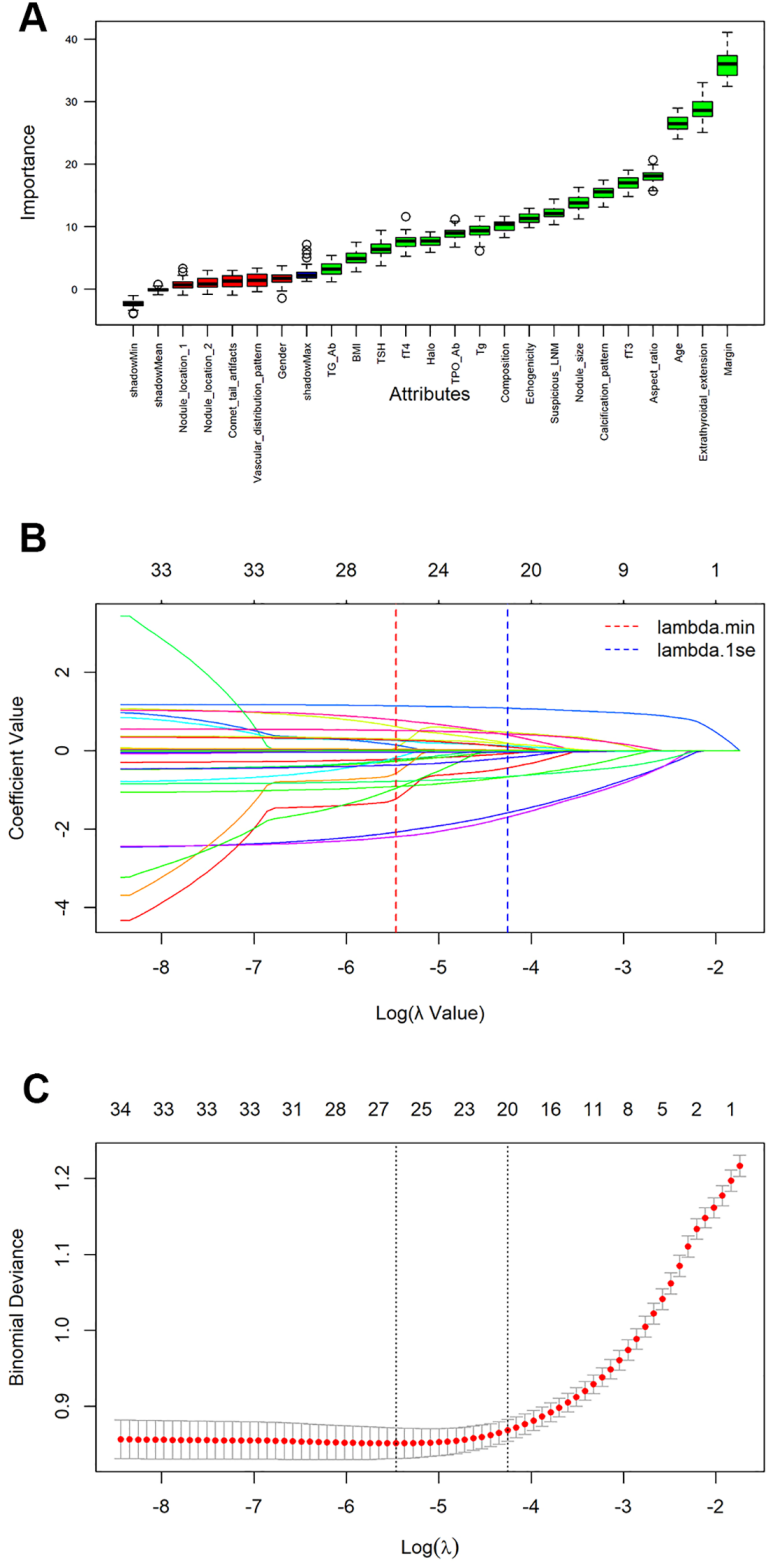
Feature selection and ranking by Random Forest and the least absolute shrinkage and selection operator (Lasso) regression analysis. **(A)** The plot shows boxplot of all the attributes plus minimum, average and maximum shadow score. Variables having boxplot in green are important variables confirmed by Random Forest. If boxplots are in red, it indicates that they were rejected. Yellow color of boxplot indicates they are tentative; **(B)** Coefficients of all variables are shrunk to 0 via Lasso regression, with stability increasing as lambda increases; **(C)** The model coefficient of λ value that minimizes the model deviation by cross-validation curve in lasso regression analysis.

The process and results of feature selection by RF are shown in **Figure 2A**. There are a total of 17 variables that serve as indicators for judging the benignity and malignancy of TNs: margin, extrathyroidal extension, age, aspect ratio, fT3, calcification pattern, nodule size, suspicious LNM, echogenicity, composition, Tg, TPO Ab, halo, fT4, TSH, BMI, Tg Ab. To further validate the outcomes of feature selection via RF, we conducted a Lasso regression analysis, as illustrated in **Figures 2B, C**. Unsurprisingly, 13 variables exactly overlapped with those confirmed in the RF analysis (margin, extrathyroidal extension, age, aspect ratio, fT3, calcification pattern, nodule size, suspicious LNM, echogenicity, composition, TPO Ab, halo, fT4). Meanwhile, Lasso regression also included variables such as nodule location 1, nodule location 2, vascular distribution pattern, and gender—all of which were excluded in RF—while excluding variables like BMI, TSH, Tg Ab, and Tg that were included in RF. Subsequently, we assessed data multicollinearity using the variance inflation factor (VIF) method. The results indicated that most variables in the model exhibited low levels of multicollinearity, with only composition and echogenicity showing mild collinearity (**Supplementary Table S1**). The above analysis indicates that the results of RF and Lasso regression were largely consistent. Given that the primary objective of subsequent model construction was to enhance prediction accuracy rather than interpretability, we selected the variables screened by RF to develop the subsequent model.

### 3.3 Development and validation of ML models

**Figure 3:**
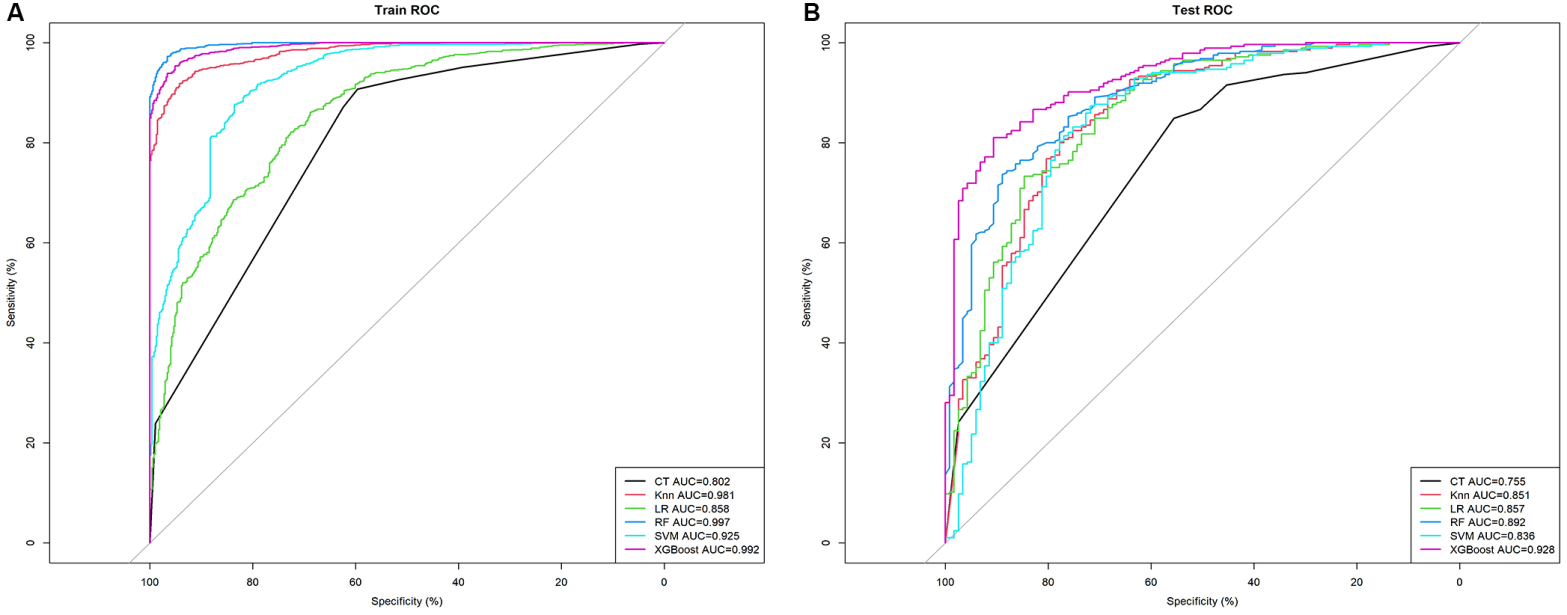
The mixed ROC curves of the six machine learning models for prediction of TNs. **(A)** The mixed ROC curves in the training cohort; **(B)** The mixed ROC curves in the test cohort. CT, classification tree; KNN, K-nearest neighbor; LR, logistic regression; RF, random forest; ROC receiver operating characteristic; SVM, support vector machine; XGB, extreme gradient boosting.

Using the selected features, we developed six models (CT, KNN, LR, XGBoost, SVM, and RF) to assess the malignant risk of TNs. The ROC curves (**Figure 3A**) and **Table 2** show that in the training set, the six models exhibited good performance, with AUC values ranging from 0.802 (CT) to 0.997 (RF), sensitivity from 0.861 to 0.978, and specificity from 0.596 to 0.966. When applied to the test data, the AUCs of the CT, KNN, RF, and SVM models dropped significantly compared to those in the training data (All *p* < 0.05 by DeLong test). In contrast, the AUCs of XGBoost (achieving an AUC of 0.928, accuracy of 0.851, sensitivity of 0.933, and specificity of 0.650) and LR remained relatively stable, indicating that these two models maintain strong robustness in internal validation (**Figure 3B**). Notably, although the AUC of RF in the test set was lower than that in the training set, it still ranked second only to XGBoost and higher than that of LR.

**Table 2:** Predictive performance comparison of the six types of machine learning algorithms in the training and test dataset.

Models	Accuracy	Sensitivity	Specificity	AUC	F1 score	PPV	NPV
Training Set							
CT	0.817	0.907	0.596	0.802	0.876	0.846	0.725
KNN	0.912	0.974	0.762	0.981	0.940	0.909	0.922
LR	0.811	0.861	0.688	0.858	0.866	0.971	0.669
RF	0.970	0.972	0.966	0.997	0.979	0.986	0.934
SVM	0.882	0.968	0.671	0.925	0.921	0.878	0.671
XGBoost	0.952	0.978	0.887	0.992	0.966	0.954	0.943
Test Set							
CT	0.761	0.867	0.503	0.755	0.837	0.810	0.608
KNN	0.836	0.937	0.590	0.851	0.890	0.848	0.793
LR	0.838	0.954	0.556	0.857	0.893	0.840	0.846
RF	0.828	0.889	0.737	0.892	0.879	0.880	0.703
SVM	0.828	0.940	0.556	0.836	0.886	0.838	0.556
XGBoost	0.851	0.933	0.650	0.928	0.899	0.866	0.800

AUC, the area under the curve; CT, classification tree; KNN, K-nearest neighbor; LR, logistic regression; RF, random forest; SVM, support vector machine; XGB, extreme gradient boosting.

**Figure 4:**
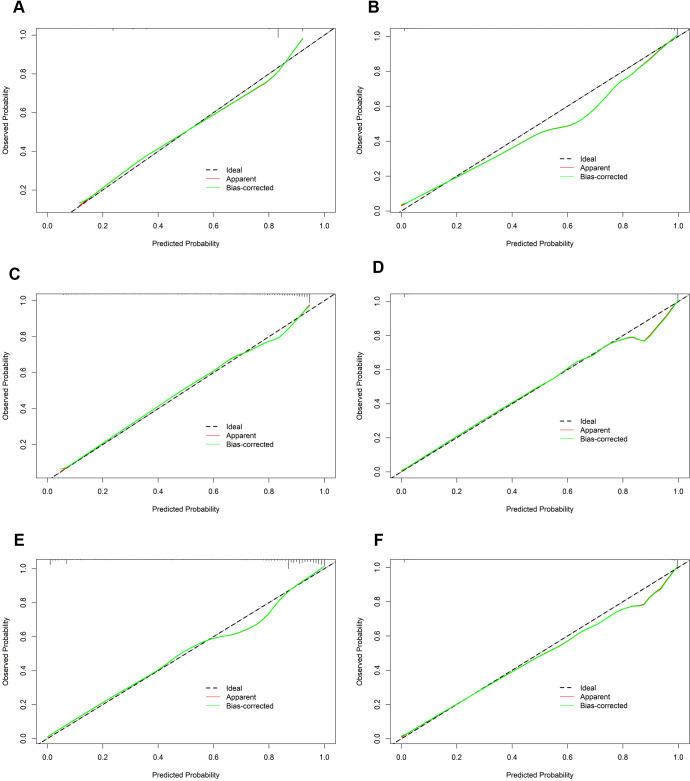
The calibration curves of the six machine learning models for prediction of TNs in the training cohort. **(A)** CT, classification tree; **(B)** KNN, K-nearest neighbor; **(C)** LR, logistic regression; **(D)** RF, random forest; **(E)** SVM, support vector machine; **(F)** XGBoost, extreme gradient boosting.

**Figure 5:**
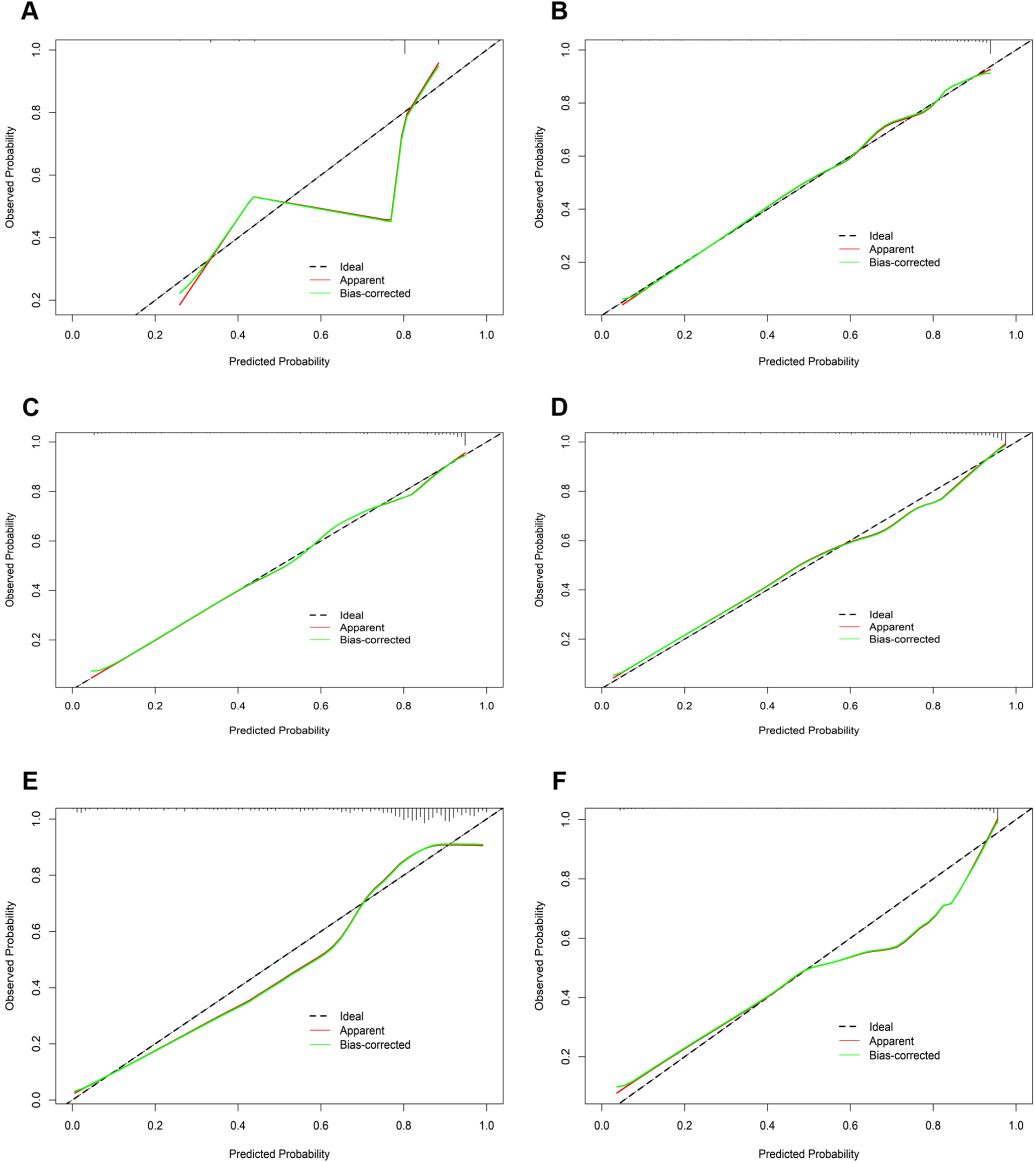
The calibration curves of the six machine learning models for prediction of TNs in the test cohort. **(A)** CT, classification tree; **(B)** KNN, K-nearest neighbor; **(C)** LR, logistic regression; **(D)** RF, random forest; **(E)** SVM, support vector machine; **(F)** XGBoost, extreme gradient boosting.

**Figure 6:**
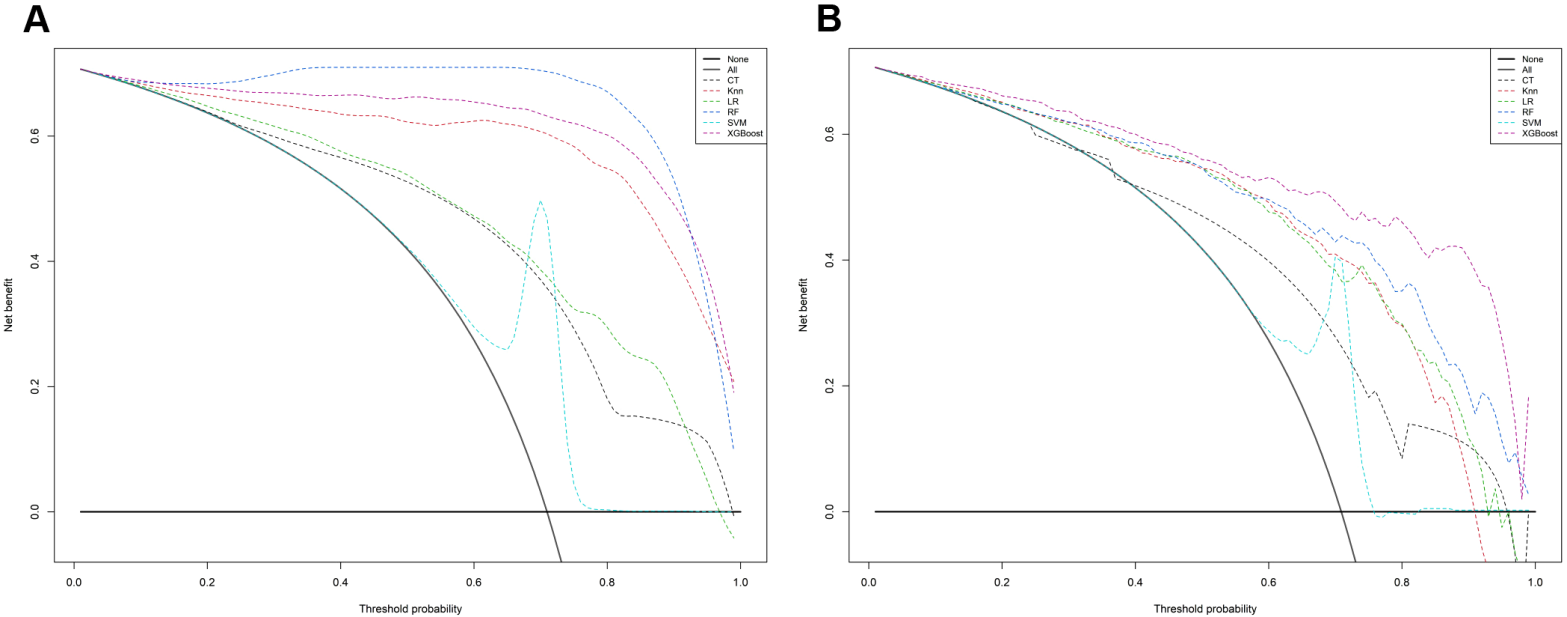
The mixed decision curves of the six machine learning models for prediction of TNs. **(A)** The mixed decision curves in the training cohort; **(B)** The mixed decision curves in the test cohort. CT, classification tree; KNN, K-nearest neighbor; LR, logistic regression; RF, random forest; ROC receiver operating characteristic; SVM, support vector machine; XGB, extreme gradient boosting.

In the calibration assessment, except for the CT model, the calibration plots for both the training (**Figures 4A–F**) and test (**Figures 5A–F**) groups showed a high degree of consistency between predicted probabilities and observed outcomes, with deviations consistently within a 10% margin of error. Analysis of the DCA plots in **Figures 6A, B** revealed that across lower risk probability thresholds, all models yielded a greater net benefit for treatment decision-making compared to the no-intervention strategy. In the 0.2 to 0.9 threshold range, all models except SVM yielded a greater net benefit compared to the reference strategy, thus providing valuable clinical insights to assist clinicians in treatment decision-making.

Next, we compared the diagnostic performance of the RF and XGBoost models in TNs. First, the Youden index was used to determine the optimal thresholds for each model (RF: 0.739; XGBoost: 0.79). After integrating all performance metrics, the Friedman test revealed a significant difference in performance between the two models (*p* = 0.0082), with the XGBoost model exhibiting superior average performance (XGBoost mean = 0.852; RF mean = 0.8052). Furthermore, the robustness of the XGBoost model was assessed by merging the training and validation set data, followed by five-fold cross-validation, which yielded an average AUC of 0.983. Lastly, the calibration and DCA curves were plotted (**Supplementary Figure S1**). Based on these results, we selected the XGBoost model as the optimal predictive model.

Furthermore, the SHapley Additive exPlanations (SHAP) method was employed to interpret the importance of variables in the model. The results, as shown in **Supplementary Figure S2**, indicate the following ranking of the top variables: margin, age, TSH, and extrathyroidal extension. Additionally, we compared the predictive performance of our XGBoost model with that of the existing ACR-TIRADS model. Compared with the XGBoost model, the ACR scoring model demonstrated inferior predictive efficacy (validation set: AUC, 0.764; Accuracy, 0.754; Sensitivity, 0.823; Specificity, 0.583; DeLong test, *p* < 0.01). Finally, to facilitate practical application, we developed a web-based calculator (accessible at https://pres-app.shinyapps.io/XGBoost/), enabling seamless integration of our findings into clinical decision-making.

## 4 Discussion

In the present study, we collected 22 common clinical indicators obtained from 2014 patients with TNs. Using Lasso regression and RF for variable screening, we found largely consistent results. The observed differences of those two methods can be attributed to Lasso prioritizing linear independent effects versus random forest modeling nonlinear interactions. As a result, 17 key feature variables were identified, which encompassed demographic data, blood biochemical indicators, and ultrasound feature outcomes. We then developed and compared six ML models, all achieving test-set accuracy and AUC above 0.75. RF and XGBoost performed best, with statistical analyses confirming XGBoost as the optimal model. Finally, to facilitate clinical translation, a web-based calculator was developed to generate personalized risk stratification (low/high risk) based on input parameters. For low-risk patients, this tool supports clinical decisions to skip FNA and adopt active surveillance, potentially optimizing patient management workflows.

Current ultrasound-based classification systems allow for more precise malignancy risk stratification in TNs (3–5). Notably, accumulating evidence shows that integrating clinical, biological, ultrasonic, and cytological features can further enhance prediction accuracy (8, 15–17). Nomograms, as visual derivatives of logistic regression, have been widely used in disease prediction to facilitate individualized risk assessment for clinicians. However, these models suffer from inherent limitations: strict assumptions (e.g., linearity, low dimensionality), challenges in capturing complex nonlinear relationships and feature interactions, poor handling of high-dimensional/multi-modal data, and suboptimal generalization ability. In contrast, ML models such as RF and XGBoost offer distinct advantages: strong nonlinear modeling capacity, adaptability to diverse data types, and the ability to automatically identify high-order feature interactions. These strengths enable them to process high-dimensional/unstructured data (e.g., images, text) and enhance generalization and robustness through ensemble strategies—key advantages for complex disease prediction and multimodal data fusion. As an open-source package, XGBoost has demonstrated exceptional and consistent performance in recent ML challenges involving disease prediction and data mining—such as predicting COVID-19 mortality risk (18) and oropharyngeal cancer recurrence risk (19), etc. In this study, the XGBoost model outperformed traditional ACR-TIRADS model in diagnostic efficacy (AUC: 0.928 vs. 0.764), highlighting its potential to enable precise TN risk stratification, reduce unnecessary invasive procedures, and serve as a scalable framework for integrating multimodal data in complex disease prediction.

Genetic testing kits (e.g., ThyroSeq V3, RosettaGXReveal) have been applied in clinical practice and exhibit excellent diagnostic value (20). However, due to their high cost and limited accessibility in many healthcare settings, genetic testing remains underutilized (21, 22). In contrast, this study exclusively incorporated readily available clinical data—achieving diagnostic performance comparable to genetic testing through AI algorithms. Of the 17 selected features, 8 were ultrasonographic characteristics, 3 were clinical demographics, and 6 were thyroid function tests. Most of these features are widely recognized by clinicians as key indicators for TN diagnosis, with nodule margin being the most influential factor, followed by extrathyroidal extension, patient age, and aspect ratio—findings consistent with our prior research (8). Notably, our ML models included thyroid function biomarkers (fT3, fT4, Tg, Tg Ab, TPO Ab, TSH), whose roles in thyroid pathology diagnosis have long been debated. While Tg and Tg Ab are traditionally used to monitor postoperative thyroid cancer recurrence, Yuxin Zheng et al. first demonstrated their utility in differentiating benign and malignant thyroid follicular tumors (23). Additionally, our previous nomogram model integrated TPO Ab as a key serological marker for TN diagnosis, underscoring the clinical value of incorporating these biomarkers into risk stratification frameworks.

In parallel, Krzysztof et al. reported that among patients with cytologically indeterminate TNs, those with malignant histopathology had significantly higher serum TSH levels than benign cases within the same cytological category (24). This finding was reinforced by a meta-analysis by Xiao-Yu Fan et al., which demonstrated that serum TSH measurement exhibits high sensitivity and specificity for diagnosing differentiated thyroid cancer (25). In contrast, Shumei Miao et al. emphasized the role of additional thyroid function parameters (Tg, fT4, fT3) in predicting malignant nodule incidence (25), while other studies argue that standard thyroid function tests lack discriminatory power for nodule malignancy (26–28).

These conflicting conclusions likely stem from heterogeneous study populations—for example, cohorts including individuals with metabolic comorbidities (e.g., insulin resistance), which may independently elevate thyroid cancer risk (29, 30). Against this context, the current study’s findings are notable: by leveraging ML, we demonstrated the ability to unravel complex data relationships through large-scale analysis—a capability unmatched by traditional statistical models. Given the ongoing complexities of thyroid disease biomarkers and potential confounding variables, further research is essential to clarify the nuanced interplay between thyroid function and cancer risk.

Several limitations must be considered when interpreting the study’s findings. First, the retrospective design inherently introduces selection bias, as historical data may lack completeness or include unmeasured confounders. Second, ultrasound features were manually extracted by sonographers rather than automatically derived from images, a process that could introduce observer variability and subjective bias during feature interpretation. Finally, training-set AUCs were higher than test-set values, indicating potential overfitting—likely due to model complexity and limited training data generalizability. To address this, we plan multicenter prospective trials to expand the sample size and simplify the model architecture, enhancing validity and external generalizability.

## 5 Conclusions

In summary, we developed six ML models integrating clinical characteristics, ultrasonographic features, and thyroid function data to predict the malignancy of TNs. Among these, the XGBoost model exhibited superior diagnostic performance, thus leading to the development of a web-based calculator—a practical tool enabling clinicians to assess the probability of TN malignancy. However, the performance of the model should be further investigated using aprospective dataset.

## Data Availability

The raw data supporting the conclusions of this article will be made available by the authors, without undue reservation.
