# Supplementary material for: XGBoost-based machine learning model combining clinical and ultrasound data for personalized prediction of thyroid nodule malignancy
Source: Front Endocrinol (Lausanne). 2025 Jul 29;16:1639639. doi: 10.3389/fendo.2025.1639639 (PMC12339320; doi:10.3389/fendo.2025.1639639)
Supplement: Supplementary file 1 [file DataSheet1.docx]

Supplementary Material

**Table S1. Generalized Variance Inflation Factor (GVIF) Statistics for Various Features**

| **Characteristics** | **GVIF** | **Df** | **GVIF^(1/(2*Df))** |
| --- | --- | --- | --- |
| Age | 1.05281 | 1 | 1.026065111 |
| BMI | 1.051273 | 1 | 1.025315965 |
| Nodule_size | 1.376724 | 1 | 1.173338616 |
| Margin | 1.096014 | 1 | 1.04690698 |
| Extrathyroidal_extension | 1.071105 | 1 | 1.034942208 |
| Halo | 1.154734 | 2 | 1.036622247 |
| Composition | 46.51093 | 3 | 1.896382081 |
| Echogenicity | 42.00862 | 3 | 1.864474304 |
| Calcification_pattern | 1.244755 | 3 | 1.037163752 |
| Suspicious_LNM | 1.036039 | 1 | 1.017859792 |
| Aspect_ratio | 1.227173 | 1 | 1.107778519 |
| TSH | 1.155215 | 1 | 1.074809347 |
| TPO_Ab | 1.165168 | 1 | 1.079429266 |
| TG_Ab | 1.135601 | 1 | 1.065645749 |
| fT4 | 1.172375 | 1 | 1.082762866 |
| fT3 | 1.148653 | 1 | 1.071752169 |
| Tg | 1.037543 | 1 | 1.018598732 |
| Nodule_location_1 | 1.378345 | 2 | 1.083526373 |
| Nodule_location_2 | 1.445311 | 3 | 1.06331073 |
| Comet_tail_artifacts | 1.199383 | 1 | 1.095163561 |
| Gender | 1.083578 | 1 | 1.040950662 |

Df: Degree of Freedom; GVIF: Generalized Variance Inflation Factor.


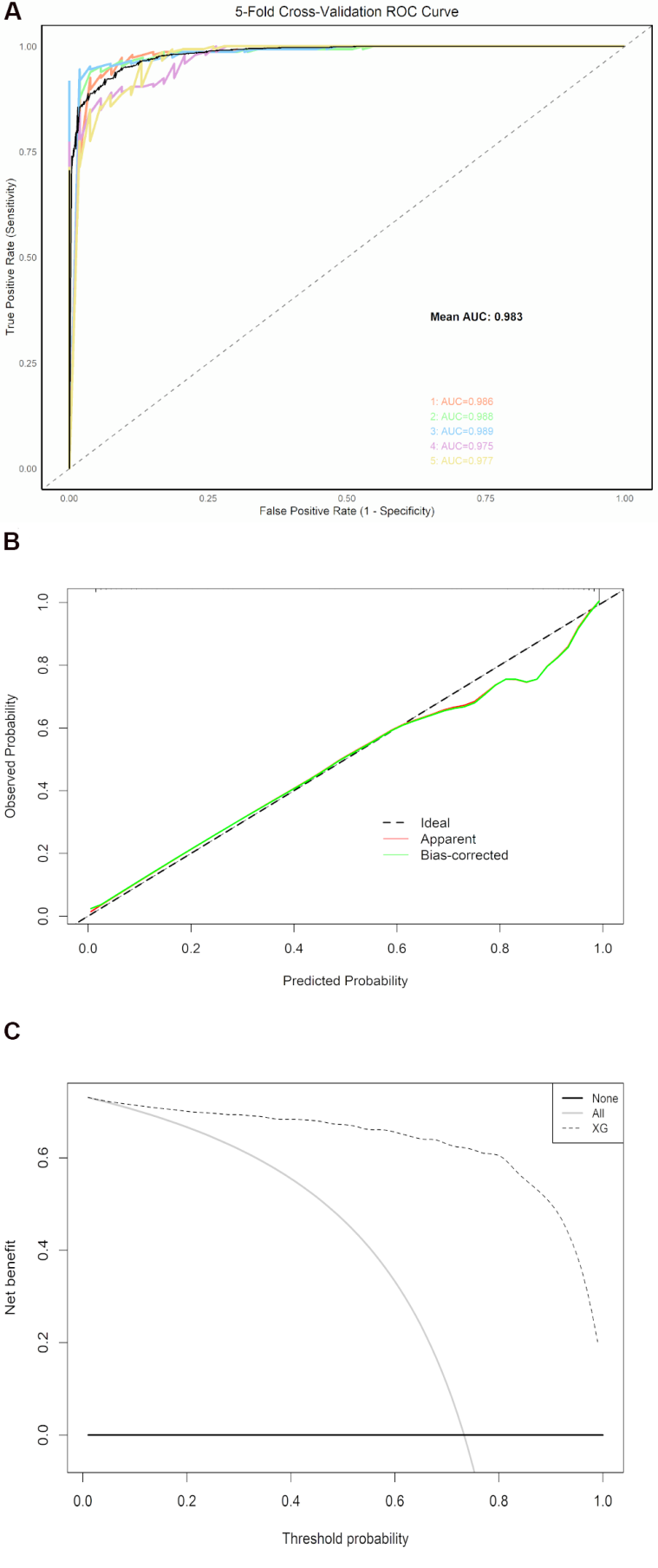


Figure S1 5 Five-fold cross-validation of the XGBoost model

(A) ROC plot of the five-fold cross-validation with an average AUC of 0.983. (B) Calibration curve of the XGBoost model. (C) DCA plot of the XGBoost model. ROC, receiver operating characteristic; AUC, area under the curve; XGBoost, extreme gradient boosting; DCA, decision curve analysis.


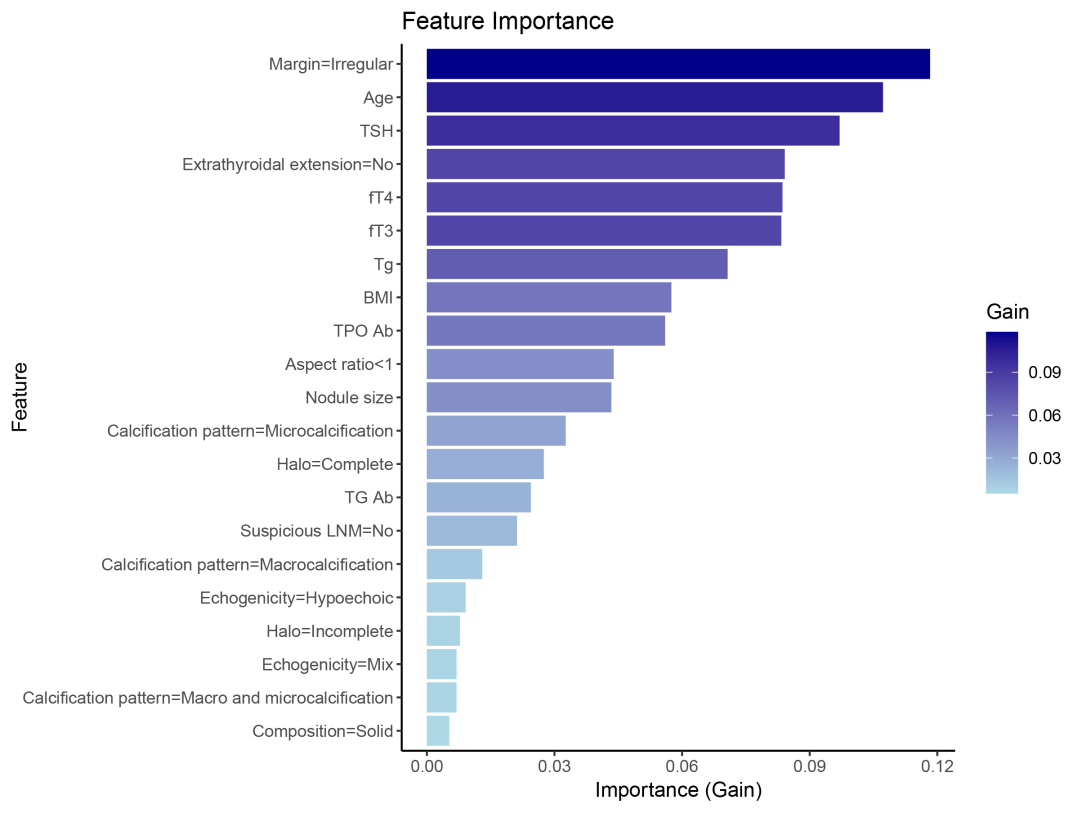


Figure S2 SHAP values of the selected features

The higher the SHAP value of each variable, the more impact and contribution to the model. SHAP, Shapley additive explanations.
